# Identifying the homology of the short human pisiform and its lost ossification center

**DOI:** 10.1186/s13227-019-0145-2

**Published:** 2019-11-25

**Authors:** Kelsey M. Kjosness, Philip L. Reno

**Affiliations:** Department of Bio-Medical Sciences, Philadelphia College of Osteopathic Medicine, 4170 City Avenue, Philadelphia, PA 19131 USA

**Keywords:** Homology, Pisiform, Calcaneus, Ossification center, Epiphysis

## Abstract

**Background:**

The pisiform and calcaneus are paralogous bones of the wrist and ankle and are the only carpal and tarsal, respectively, to develop from two ossification centers with an associated growth plate in mammals. Human pisiforms and calcanei have undergone drastic evolutionary changes since our last common ancestor with chimpanzees and bonobos. The human pisiform is truncated and has lost an ossification center with the associated growth plate, while the human calcaneus has expanded and retained two ossification centers and a growth plate. Mammalian pisiforms represent a wide range of morphologies but extremely short pisiforms are rare and ossification center loss is even rarer. This raises the question of whether the sole human pisiform ossification center is homologous to the primary center or the secondary center of other species. We performed an ontogenetic study of pisiform and calcaneus ossification patterns and timing in macaques, apes, and humans (*n* = 907) from museum skeletal collections to address this question.

**Results:**

Human pisiforms ossify irregularly and lack characteristic features of other primates while they develop. Pisiform primary and secondary center ossification timing typically matches that of the calcaneus of non-human primates, while the human pisiform corresponds with calcaneal secondary center ossification. Finally, human pisiforms ossify at the same dental stages as pisiform and calcaneal secondary centers in other hominoids.

**Conclusions:**

These data indicate that the human pisiform is homologous to the pisiform epiphysis of other species, and that humans have lost a primary ossification center and associated growth plate while retaining ossification timing of the secondary center. This represents an exceptional evolutionary event and demonstrates a profound developmental change in the human wrist that is unusual not only among primates, but among mammals.

## Background

The pisiform is an elongated, rod-shaped bone in the proximal carpal row that forms a rigid articulation between the triquetral and ulnar styloid process in almost all mammals [1, 2]. It serves as an attachment for the tendon of the *flexor carpi ulnaris* muscle and is the only carpal to possess two ossification centers with an associated growth plate [3–5]. The mammalian pisiform is functionally analogous to the calcaneus which articulates firmly to the talus and navicular and whose tuberosity is the insertion point for the calcaneal tendon. The calcaneus has been described as developing from two distinct chondrifications and being the developmental equivalent to a fusion of the pisiform and triquetral in the forelimb [6]. This relationship is further supported by morphological changes in mice with altered *Pitx1* expression whereby misexpression in the forelimb produces fusion of the pisiform and triquetral into a calcaneus-like structure [7]. The pisiform and calcaneus also fall within similar *Hox* gene expression territories during limb development [8]. Furthermore, the pisiform and calcaneus are the only carpals or tarsals to possess two ossification centers with an associated growth plate [4]. These functional, developmental, and embryological similarities indicate that the calcaneus is likely paralogous to the pisiform and triquetral in the forelimb [6].

**Fig. 1:**
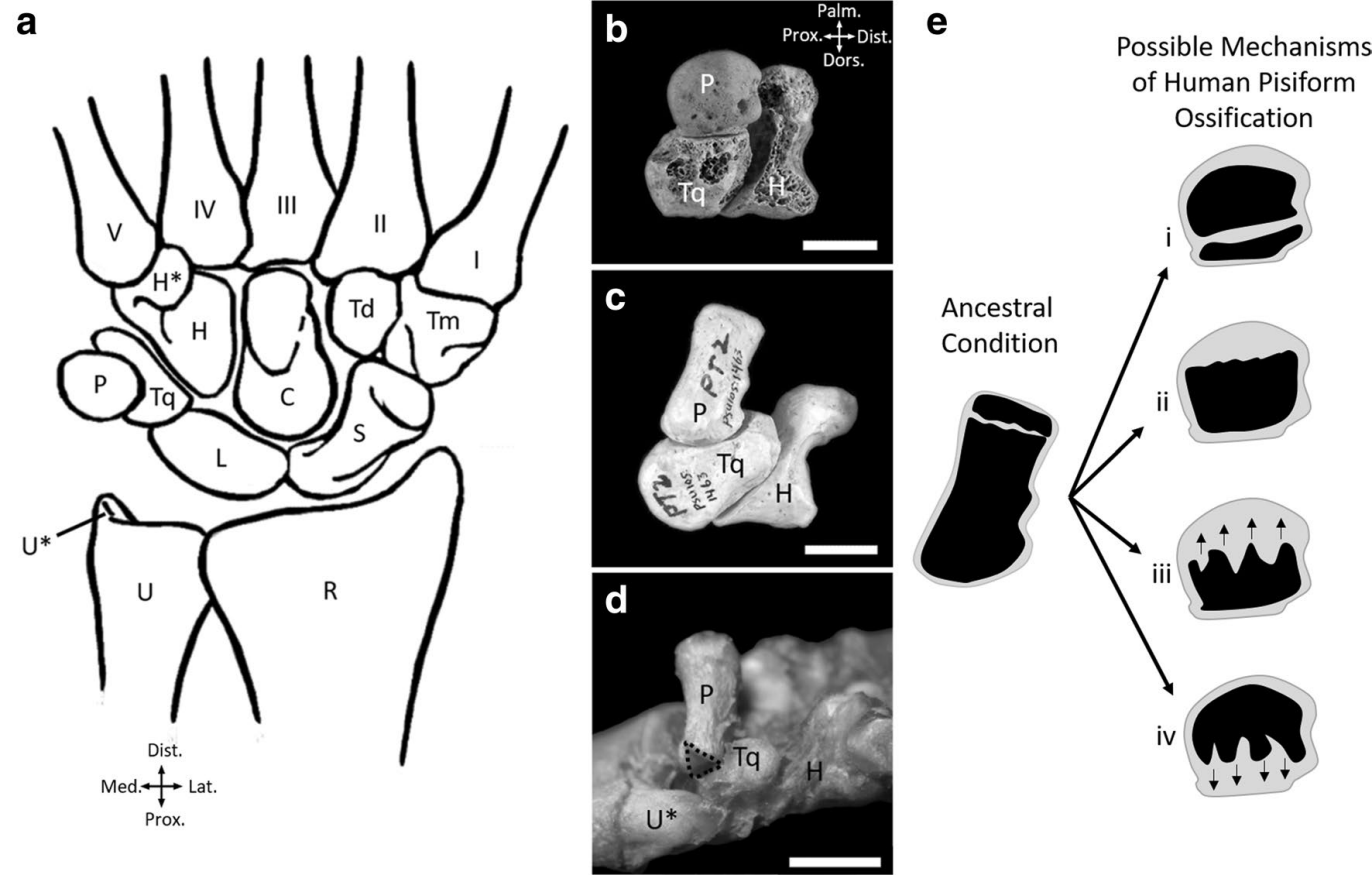
Wrist anatomy and hypotheses of human pisiform ossification. **a** Carpal configuration of the human wrist (palmar view). **b**–**d** Ulnar view showing pisiform shape, projection, and articulations in human (**b**), chimpanzee (**c**), and macaque (**d**). Palmar is up, dorsal is down. The pisiform articulates only with the triquetral in humans and chimpanzees while it articulates with both the triquetral and ulnar styloid process in macaques (dashed line shows ulna articular surface of the pisiform). The human pisiform is pea-shaped with minimal projection beyond the hamate, while both chimpanzees and macaques have a rod-shaped pisiform with palmar projection beyond the hamate. Abbreviations: metacarpals (numbered I–V), capitate (C), hamate (H), hamate hamulus (H*), lunate (L), pisiform (P), radius (R), scaphoid (S), trapezoid (Td), trapezium (Tm), triquetral (Tq), ulna (U), ulnar styloid process (U*). Scale bars = 1 cm. **e** Hypotheses for possible mechanisms underlying human pisiform reduction. Cartilage is gray and bone is black. The ancestral condition represents a primary ossification center with one secondary ossification center on the palmar side of the bone with a growth plate between. Four possible mechanisms for human pisiform ossification are: (i) early fusion of two ossification centers; however, regular development of two ossification centers has not been documented in humans, (ii) failure to form a secondary ossification center within the cartilaginous epiphysis, but maintenance of a growth plate and subchondral surface, (iii) loss of the secondary ossification center with direct invasion from the primary center toward the palmar end of the bone, or (iv) loss of the primary ossification center with direct invasion from the epiphysis toward the dorsal end of the bone. Arrows within pisiform cartilage indicate an advancing ossification front

The pisiform and calcaneus have both undergone substantial evolutionary changes in hominoids (ape and humans) associated with the evolution of novel locomotor patterns [9], the most drastic of which occurs in humans. The human wrist comprises eight carpal bones, arranged into two rows. The proximal row contains the scaphoid, lunate, triquetral, and pisiform, while the distal row contains the trapezium, trapezoid, capitate, and hamate (Fig. 1) [5]. This configuration is common among mammals, although the number of individual carpal bones can vary [10]. In most mammals, including monkeys, the pisiform forms a rigid articulation with the triquetral and styloid process of the ulna (Fig. 1d), limiting ulnar deviation (bending the wrist toward the ulnar side). Hominoid wrists are characterized by proximal retreat of the distal ulna, whereby the ulnar styloid process has lost its articulation with the pisiform and triquetral, allowing for greater ulnar deviation [9, 11]. Hylobatid (gibbons and siamangs) pisiforms are supported proximally by a novel ossification within the meniscus called the *os Daubentonii*. In contrast, orangutan pisiforms are commonly stabilized by an articulation with the hamate hamulus [12–14]. Human and African ape pisiforms articulate solely with the triquetral. This makes human, and possibly African ape, pisiforms unusual in their opportunity for proximodistal sliding mobility [12, 13, 15, 16]. While most hominoids retain the elongated pisiforms typical of mammals, orangutan pisiforms are usually short and human pisiforms are extremely truncated, producing only a “pea-shaped” nubbin of bone (Fig. 1a, b). The functional implications of pisiform reduction are not well understood.

While the pisiform has become reduced in the course of human evolution, the human calcaneus is wider and more robust than in apes. An expanded calcaneal tuber has resulted in prominent lateral and medial plantar cornua [17] and human calcanei have a vertical longitudinal axis compared to the angled one in apes [18]. The human calcaneus differs from great apes not only in its overall shape, but also in its skeletal composition. Great ape calcanei have a thick outer cortical shell while human calcanei are remarkably thin with expanded trabecular bone volume. These features in the human facilitate energy dissipation during heel strike in the course of bipedal locomotion [17].

The postcranial skeleton, including carpals and tarsals, develops via endochondral ossification [19]. During endochondral ossification, initial skeletal condensations made of mesenchymal cells differentiate into chondrocytes. The chondrocytes at the center of the cartilage model organize into columns and begin to undergo the sequential process of proliferation, hypertrophy (with matrix production), and ultimately cell death. This process then proceeds toward each end of the anlagen. The perichondrium at the middle of the cartilage model matures into periosteum and begins to form a bone collar encircling the hypertrophic chondrocytes. At this point, a periosteal bud invades the model providing an arterial supply to the center of the element. This opens a conduit by which osteoclasts and osteoblasts enter the cartilage model and replace the calcified matrix with bone, thus forming the primary center of ossification [19].

In a typical long bone, the progression of the maturing chondrocytes is arrested prior to reaching either end, forming a growth plate with three characteristic zones: reserve, columnar, and hypertrophic [20, 21]. Reserve zone chondrocytes provide a population of progenitor cells for continued growth and also organize the proliferating chondrocytes into longitudinal columns, which are maintained until they undergo apoptosis [20]. This growth plate is responsible for longitudinal growth via deposition of new bone at the interface between hypertrophic chondrocytes and the ossification front of the primary ossification center. The relatively undifferentiated hyaline cartilage lying at the extremities of the bone beyond each reserve zone is the epiphysis. In mammals and lizards (Lacertilia), the cartilaginous epiphysis will undergo secondary ossification radially from its center later in ontogeny in a manner similar to the short bones of the wrist and ankle that typically lack growth plates [19, 21–28]. This differs from what is observed in most bird, dinosaur, chelonian, and crocodilian epiphyses which do not form secondary centers of ossification and thus remain cartilaginous beyond the growth plate [22–24, 29]. As the mammalian secondary ossification expands, the growth plate is maintained between the primary ossification center and bony epiphysis while longitudinal growth continues and the two centers fuse upon cessation of growth [30].

Growth plates clearly vary in their rates of growth between different cites of the skeleton and between species [31–36]. Beyond variations in growth rate, the gain and loss of growth plates are viable mechanisms of evolutionary change. Unlike other long bones, mammalian metacarpals and metatarsals only form a growth plate and secondary center of ossification at one end. The opposite end undergoes direct ossification, where the columnar and hypertrophic zones disorganize and are overcome by the trailing primary center of ossification front that invades the epiphysis directly [37–40].

Major ossification changes accompany the evolutionary changes observed in human pisiforms, which develop from a single ossification center, indicating that truncation occurred through the loss of one ossification center and the associated growth plate [4]. A rod-shaped pisiform is present in the hominin ancestor *Australopithecus afarensis* (AL 333-91) at ~ 3.2 Ma [41], revealing that truncation of this bone is a recent evolutionary event. This highly unusual morphology raises interesting developmental and evolutionary questions. First, which structures are lost from the human pisiform and what is the homologous relationship of the remaining human pisiform to the human calcaneus and pisiforms of other primates? Its current morphology presents a few possibilities. One is that the growth plate fuses early (Fig. 1e–i), but we would expect the regular formation of two ossification centers, which has not been documented in humans [42–45]. Another possibility is that the secondary center fails to ossify as occurs in birds and crocodiles (Fig. 1e-ii). We would expect ossification to initiate in the dorsal end of the pisiform, closest to the triquetral, but with the subchondral surface typically underlying a growth plate at the palmar end. Another possibility is that the growth plate has simply been lost due to altered cartilage patterning during limb development. If this is the case, then the primary center of ossification could invade directly into the epiphysis as occurs in mammalian metacarpals and metatarsals (Fig. 1e-iii). We would expect the initial appearance and timing of pisiform ossification to resemble that of other mammals, particularly hominoids. Last, it is possible that a novel evolutionary change has occurred in that the primary center of ossification and growth plate have both been eliminated. In this case, we would expect the timing and appearance of human ossification to resemble that of a secondary center of ossification in the human calcaneus and pisiforms of other hominoids (Fig. 1e-iv).

A second issue is whether changes in pisiform ossification timing coincide with calcaneus ossification changes in humans. During embryogenesis, the forelimb and hind limb share common expression patterns and functions of many developmental genes [46]. These shared genetic networks have the potential to produce developmental constraints and subsequent high levels of morphological integration (covariation) between the limbs [47]. Such a dramatic change in human pisiform ossification may have correlated effects in the calcaneus, in which case we would expect the progression or relative timing of ossification to differ from closely related taxa. However, both the pisiform and calcaneus have undergone dramatic changes in morphology in the course of human evolution. The forelimb and hind limb are differentiated by the action of limb identity transcription factors, *Tbx5* verses *Pitx1* and *Tbx4*, respectively [46, 48]. Thus, the regulatory potential may exist to enable these homologous bones to evolve diverging morphologies and ossification patterns without consequence. If so, the loss of an ossification center and growth plate in the pisiform will occur independently of any changes in the calcaneus.

Previous studies of carpal and tarsal development in hominoids have reported almost exclusively on primary center ossification and with no mention of the calcaneal and pisiform epiphyses. The pisiform is the last or second-to-last carpal to begin ossification in chimpanzees, orangutans, and humans and the fifth of nine carpals in macaques, while the calcaneus is the first tarsal to begin ossification in humans, apes, and monkeys [49–52]. Human pisiform ossification begins between 9 and 12 years of age [5]. This appears to be later in development than the primary ossification center of chimpanzees or gorillas; however, the comparative ossification timing across species and relationship between the primary and secondary centers remain unknown [4]. We address these questions of human pisiform homology and their potential coordinated evolution with the human calcaneus through a comparative analysis of the ossification of these bones. We compare the morphological progression of pisiform primary and secondary center ossification of humans, apes, and macaques to developmental series established in mice. In addition, we conducted a comprehensive analysis of the timing of pisiform and calcaneus ossification and dental eruption patterns in macaques, apes, and humans.

## Results

### Human pisiform ossification resembles an epiphysis

We surveyed museum skeletal collections to determine dental eruption and ossification stage of pisiforms and calcanei in juvenile humans (*Homo sapiens*), chimpanzees (*Pan troglodytes*), bonobos (*Pan paniscus*), gorillas (*Gorilla* sp.), orangutans (*Pongo* sp.), hylobatids (*Hoolock* sp., *Hylobates* sp., and *Nomascus* sp. [53] and *Symphalangus syndactylus*), and macaques (*Macaca* sp.) (*n* = 907, Table 1). This sample represents the full range of pisiform and calcaneus postnatal development. We observed fully cartilaginous pisiforms (see example in Fig. 3) in some well-preserved chimpanzee, gorilla, orangutan, hylobatid, and human juveniles with deciduous dentition (*n* = 27). Very few bonobo specimens with both deciduous dentition and preserved pisiforms were available (*n* = 5), and macaque pisiforms begin to ossify during fetal development [50]; therefore, we did not observe cartilaginous pisiforms in either of these groups. All calcanei had at least a primary ossification center since ossification begins during fetal development in all surveyed groups [49–52]. Mouse specimens were analyzed histologically using Safranin-O and Fast Green stain and microCT scans.

**Table 1:** Sample sizes and data subsets

Taxonomic group	All	Pisiform-calcaneus	Pisiform-dentition	Calcaneus-dentition
Human	83	48	45	80
Chimpanzee	281	190	188	273
Bonobo	39	23	22	38
Gorilla	203	124	133	185
Orangutan	74	54	56	70
Gibbon and Siamang	88	49	73	64
Macaque	139	134	129	134
Total	907	622	646	844

Sample sizes by taxonomic group for all specimens and subsets of specimens by available skeletal material. Skeletal specimens did not always preserve a pisiform, calcaneus, and dentition. Data were collected from specimens with at least two of the three skeletal elements and recorded into data subsets based on the material present. Individuals were included in all data subsets for which material was available.

In the course of endochondral ossification, bone cells invaded the initially cartilaginous models at the periosteal bud to produce the primary center of ossification. In typical long bones, the ossification proceeds towards each end until it reaches the growth plate where cartilage replacement is matched by the rate of cartilage growth. Subsequently, a second invasion within the cartilaginous epiphysis forms the secondary centers of ossification beyond the growth plate. In typical short bones, such as the majority of the carpals and tarsals, the primary center of ossification proceeds directly to the subchondral articular surfaces [54]. Mammalian calcanei are a well-known exception, having two ossification centers and a growth plate.

**Fig. 2:**
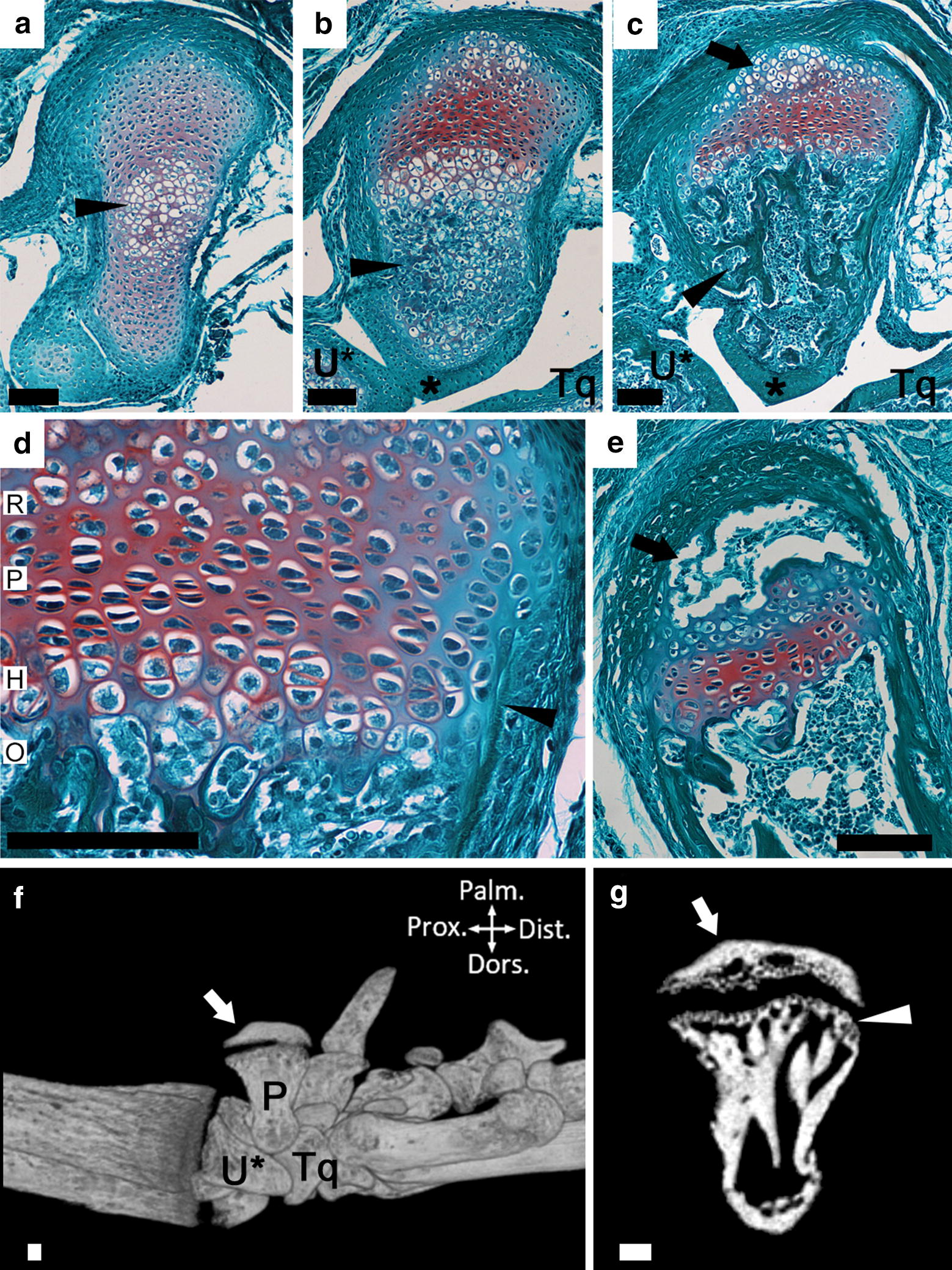
Progression of mouse pisiform ossification. **a** Histological section of early stages (P4) of primary ossification. Note the hypertrophic chondrocytes (arrowhead) at the center of the cartilage model. **b** At P6, primary ossification (arrowhead) expands towards the dorsal end where an articular surface (*) with the ulnar styloid process (U*) and triquetral (Tq) has formed. The growth plate is forming at the palmar end (top). **c** At P11, primary ossification has completed at the dorsal end and is filled with trabecular bone (arrowhead). Early stages of epiphysis formation are present (arrow). **d** At P11, the individual reserve (R), proliferative (P), and hypertrophic (H) zones and following ossification front (O) can be identified. The bone collar with adjacent perichondrial ring flanking the growth plate is identified (arrowhead). A subchondral surface is located at the boundary of hypertrophic chondrocytes and the ossification front. **e** At P23, the pisiform epiphysis is ossified (arrow). **f** Medial view of a 1 month mouse wrist visualized by microCT. The pisiform (P), ulnar styloid process (U*) and triquetral (Tq) are indicated. The ossified pisiform epiphysis remains unfused at 1 month (arrow). **g** Slice through 1-month-old mouse pisiform shows the ossified epiphysis (arrow) and subchondral surface of primary ossification (arrowhead). In all panels, palmar is up, dorsal is down, proximal is left, and distal is right. Scale bars = 100 μm

We previously established in mice that the pisiform follows an ossification pattern more typical of the calcaneus and long bones with the primary ossification center preceding the appearance of a secondary center, separated by a growth plate [4, 8]. In order to compare pisiform ossification between mice and primates, we studied the progression of mouse pisiform ossification in detail. The earliest stages of primary center ossification occur at postnatal day 4 (P4) with the appearance of hypertrophic chondrocytes (Fig. 2a). Most of the dorsal end is ossified by P6 and forms distinct articular surfaces for the triquetral and ulna (Fig. 2b). A growth plate is present at the palmar end by P6. By P11 the dorsal end is fully ossified and the palmar end maintains a well-formed growth plate with an identifiable perichondrial ring, bone collar, subchondral surface, and early stages of epiphysis ossification (Fig. 2c, d). Epiphysis ossification expands and the primary center preserves a distinct subchondral surface until at least P30 (Fig. 2e–g). Fusion occurs by 8 weeks of age (not shown).

**Fig. 3:**
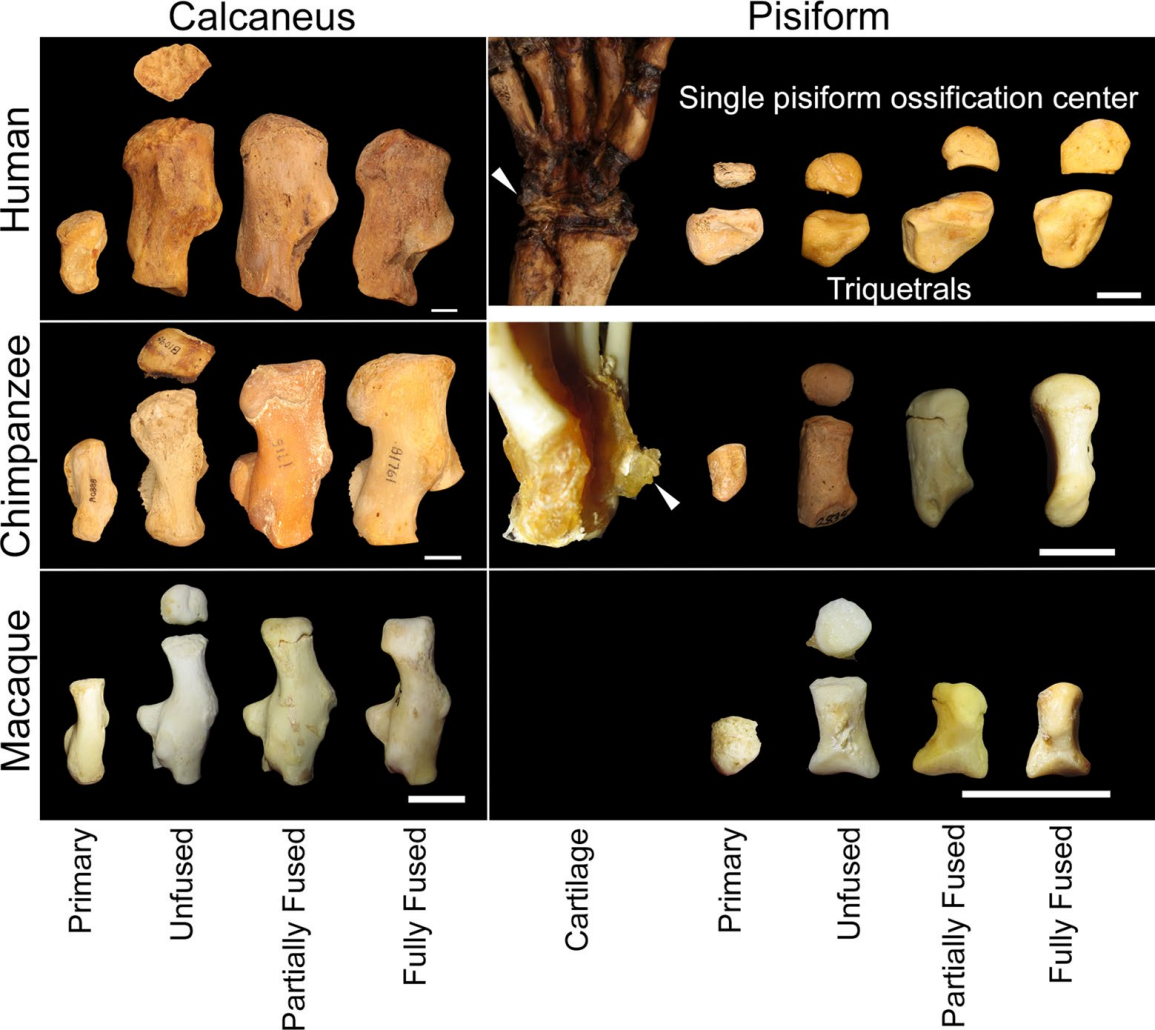
Comparison of pisiform and calcaneus ossification patterns in humans, chimpanzees, and macaques. All calcanei and non-human pisiforms progress through the same ossification stages: primary, unfused, partially fused, and fully fused. Human pisiforms develop from a single ossification center. Fully cartilaginous pisiforms were identified in some species, including humans and chimpanzees (arrowheads), but not macaques. Palmar is up, dorsal is down. Scale bars = 1 cm

**Fig. 4:**
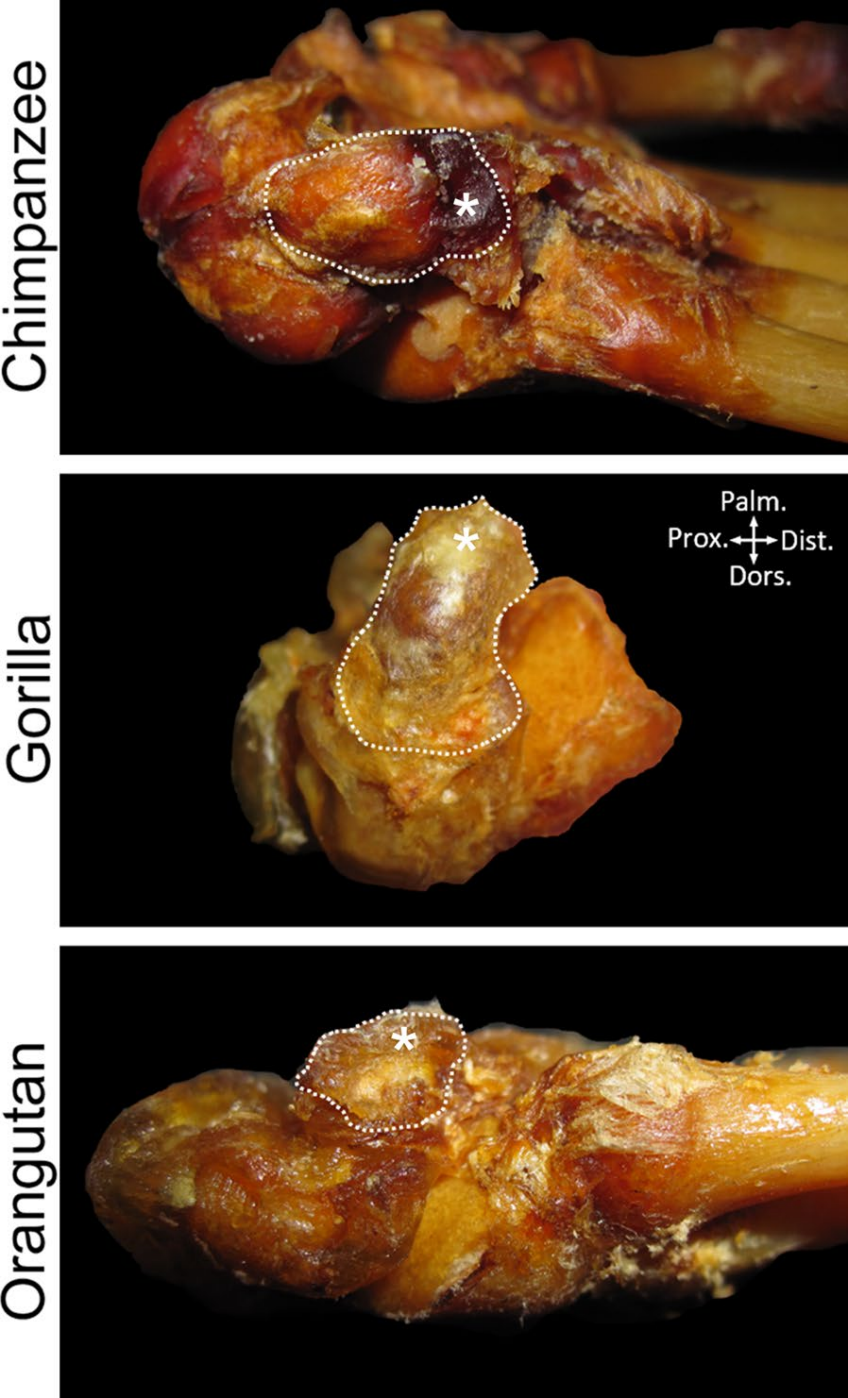
Early ossification of the proximal pisiform with epiphyseal cartilage in non-human primates. Chimpanzee, gorilla, and orangutan early pisiform primary ossification centers within preserved cartilage (white dotted outline). Primary ossification centers are located dorsally within the cartilage model and within close proximity to the triquetral. Cartilaginous epiphyses (asterisk) have not yet started secondary ossification

**Fig. 5:**
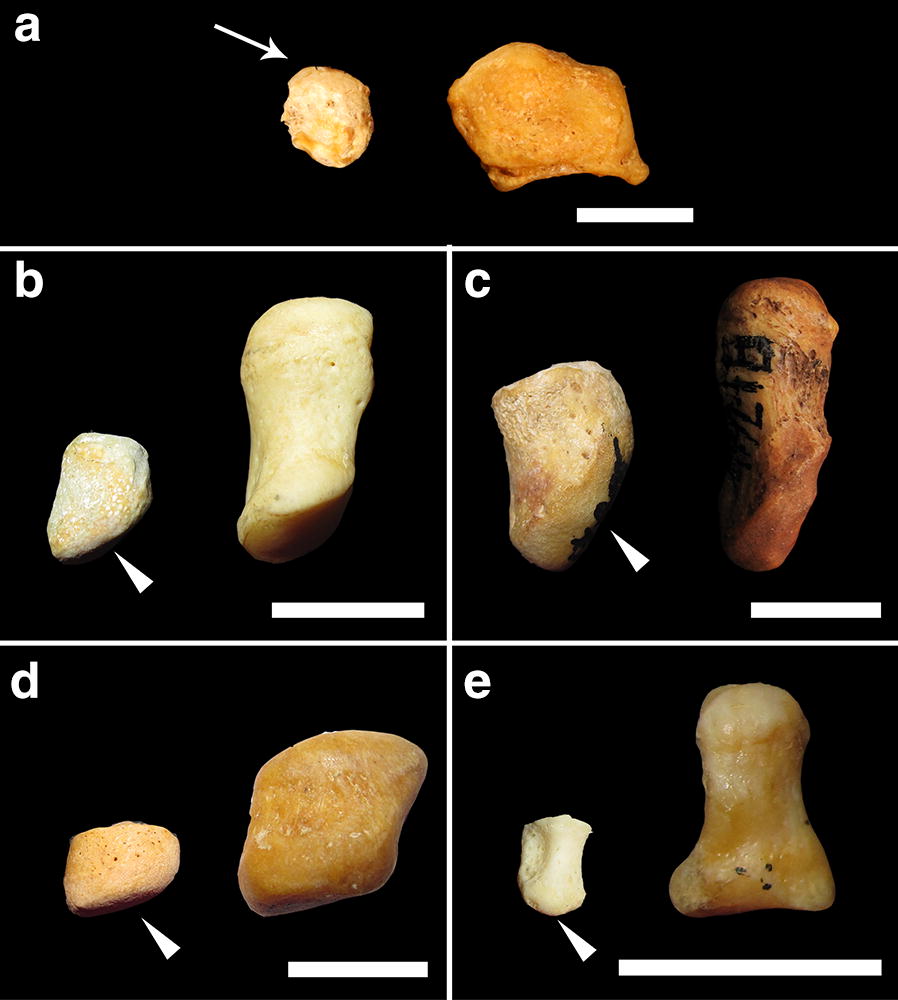
Comparison of pisiform ossification progression in humans, apes, and macaques. Progression of pisiform ossification in humans **a**, chimpanzees **b**, gorillas **c**, orangutans **d**, and macaques **e**. Early primary ossifications (Left) and adult morphology (Right) for each species. **a** Early human pisiform ossification begins at the distal/palmar end (arrow) and does not form a distinct triquetral surface until late in ossification. **b**–**e** All non-human primate pisiforms developed a distinct triquetral articular surface during the earliest stages of ossification (arrowhead). Palmar is up and dorsal is down in all panels. Scale bars = 1 cm

**Fig. 6:**
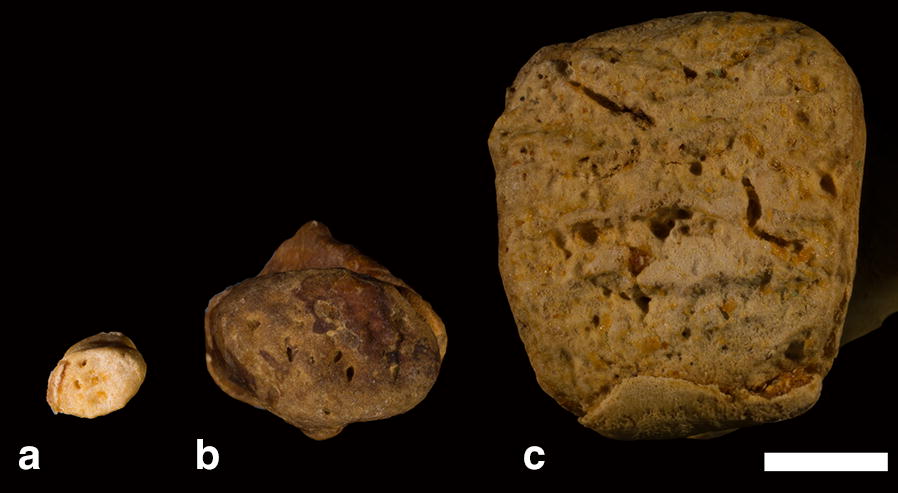
Pisiform and calcaneus subchondral surfaces. Subchondral surfaces on the distal primary ossification center in a chimpanzee unfused pisiform (left), chimpanzee unfused calcaneus (middle), and human unfused calcaneus (right). Scale bar = 1 cm

Within the primate sample, ossifying pisiforms in non-human groups and calcanei in all groups, including humans, show a similar developmental trajectory to mice (Fig. 3). Ossification of the pisiform primary center begins at the dorsal end, forming a distinct articular surface for the triquetral in early ossification (Figs. 4, 5b–e). The palmar end of the primary ossifications have a subchondral surface typical of long bones and indicative of ossification proceeding dorsally to the growth plate (Fig. 6). In contrast, the single human pisiform ossification center does not have the distinct characteristics of development observed in non-human primates or mice (Fig. 3). The early human pisiform ossification is irregularly shaped. The articular surface for the triquetral is not distinguishable early in ossification and remains poorly defined until considerably later compared to other taxa (Figs. 3, 5a). This is consistent with normal radiological findings that pisiforms in human children ossify irregularly, have a large gap between the early ossifications and the triquetral surface, and appear rounded on the palmar end [42–45]. In fact, it is the dorsal surface that appears to contain the advancing ossification front, unlike the pisiforms of the other primates and mice. This suggests that unlike most other mammals, the human pisiform begins ossification at the palmar end and progress dorsally. Such a pattern is more similar to what we would expect for epiphysis ossification.

### Human pisiform ossification corresponds to formation of calcaneal and pisiform secondary ossification

**Fig. 7:**
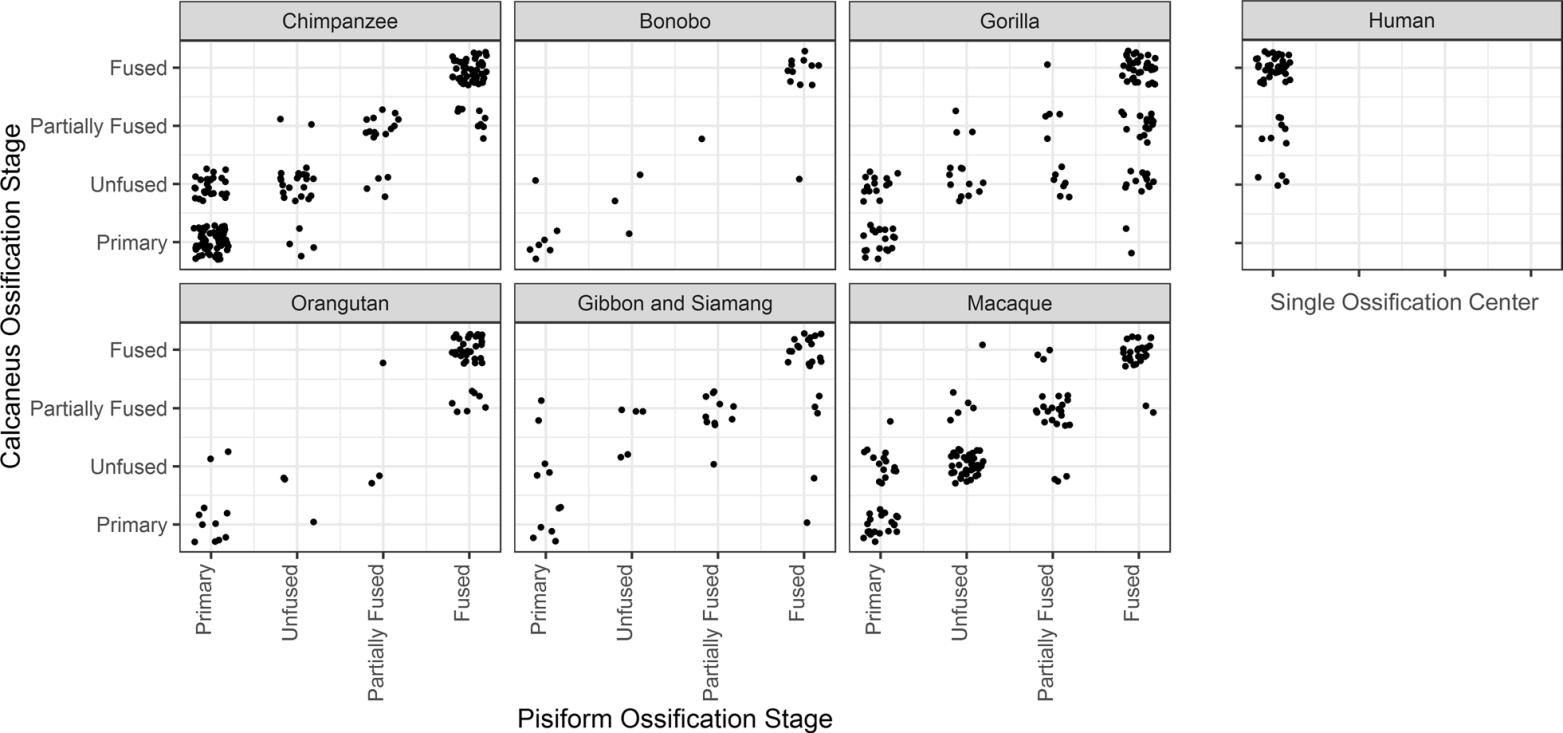
Chart of pisiform and calcaneus ossification stages for individuals within each taxonomic group. Pisiforms and calcanei occur at the same ossification stage in most non-human individuals. The single human pisiform ossification center is found corresponding only to unfused, partially fused, and fully fused calcanei

While the ossifying human pisiform is visually similar to bony epiphyses of other mammals in this study, we sought to determine if the timing of human pisiform ossification corresponds to the formation of the mammalian primary or secondary center. To accomplish this, we determined the ossification timing of the pisiform in humans, apes, and macaques relative to the calcaneus. We first categorized each calcaneus and non-human pisiform into progressive ossification stages: presence of a primary ossification only, presence of an unfused secondary center, partial fusion of the secondary center, or full fusion of the secondary center. Fully cartilaginous pisiforms were omitted from this sample. We calculated a Kendall’s tau-b correlation between pisiform and calcaneus ossification stage for non-human primates with at least one preserved calcaneus and one preserved pisiform. Ossification stages between these two bones have a significant positive correlation (*τ*_b_ = 0.806, *p* < 0.001, *n* = 574), and this relationship is maintained within each taxonomic group (*p* < 0.001, Table 2). The lowest correlation occurred in gorillas (*τ*_b_ = 0.676, *p* < 0.001, *n* = 124), whose calcaneal ossification stages were particularly variable in specimens with fully fused pisiforms (Fig. 7).

**Table 2:** Tau-b correlations by taxonomic group between pisiform ossification stage and calcaneus ossification stage

Taxonomic group	Sample size (*N*)	Correlation coefficient (*τ*_b_)	Significant (*p*)
Chimpanzee	190	0.845	< 0.001
Bonobo	23	0.886	< 0.001
Gorilla	124	0.676	< 0.001
Orangutan	54	0.804	< 0.001
Gibbon and Siamang	49	0.732	< 0.001
Macaque	134	0.839	< 0.001
Total	574		

Pisiform ossification stage and calcaneus ossification stage are highly correlated and significant in all apes and macaques

When compared across all non-human primates in this sample, we identified pisiforms and calcanei at the same ossification stage in 72.1% of individuals. Unfused calcanei are associated with primary (36.0%) and unfused (45.6%) pisiforms in all non-human groups, indicating that either the calcaneal epiphysis begins to ossify prior to the pisiform epiphysis, early pisiform secondary ossifications are not well preserved in museum collections, or both. We suspect that unfused pisiform secondary centers were sometimes not preserved and are underrepresented in this sample, thus inflating the number of primary centers identified without unfused secondary centers. Calcaneal primary ossification centers begin to form prior to that of the pisiform, so a similar pattern in the secondary center would not be surprising; however, 79.4% of individuals with unfused pisiforms (*n* = 97) also have unfused calcanei, 67.6% of partially fused pisiforms (*n* = 68) correspond to partially fused calcanei, and 75.7% of fully fused pisiforms (*n* = 222) correspond to fully fused calcanei (Fig. 7). These data indicate that there is substantial overlap in the timing of pisiform and calcaneus ossification within individuals. This association is particularly strong between unfused pisiforms and calcanei, which represents epiphysis ossification.

Human pisiform ossification centers from museum skeletal collections (*n* = 48, Table 1) corresponded to unfused, partially fused, and fully fused calcanei (Fig. 7). We did not identify any human pisiform ossifications that corresponded to a calcaneus with only a primary ossification center. These results indicate that the pisiform typically achieves a similar developmental stage as the calcaneus in non-human primates, while the single human pisiform ossification center corresponds to later stages of calcaneus development and epiphysis ossification and fusion. This suggests that the human pisiform most closely follows the ontogeny of the non-human primate pisiform epiphysis ossification and not the primary ossification center.

### Human pisiform ossification is delayed relative to other hominoids

While the pisiform and calcaneal epiphyses of non-human primates typically ossify at the same time within individuals, it is unclear whether ossification and fusion are occurring at similar ages between taxa or how human ossification timing compares to other primates. For example, given the unique morphologies of the human pisiform and calcaneus, it is possible that the ossification of both are delayed. To address this, we assessed relative age of pisiform and calcaneus development between taxa by comparing adult molar and canine eruption patterns with pisiform (*n* = 646) and calcaneus (*n* = 844) ossification stages. Adult molars (M1, M2, and M3) were considered to be erupting if any part of a cusp projected above the alveolar surface, and erupted when the entire enamel crown was above the alveolar surface and the occlusal surface was flush with the adjacent, fully erupted tooth [55, 56]. We classified dentition as deciduous if M1 had not started to erupt. Additionally, adult canine eruption was noted if M3 was fully erupted but the adult canines were not, resulting in the following classifications: deciduous, M1 erupting, M1 erupted, M2 erupting, M2 erupted, M3/canines erupting, and M3 erupted (Adult dentition).

**Fig. 8:**
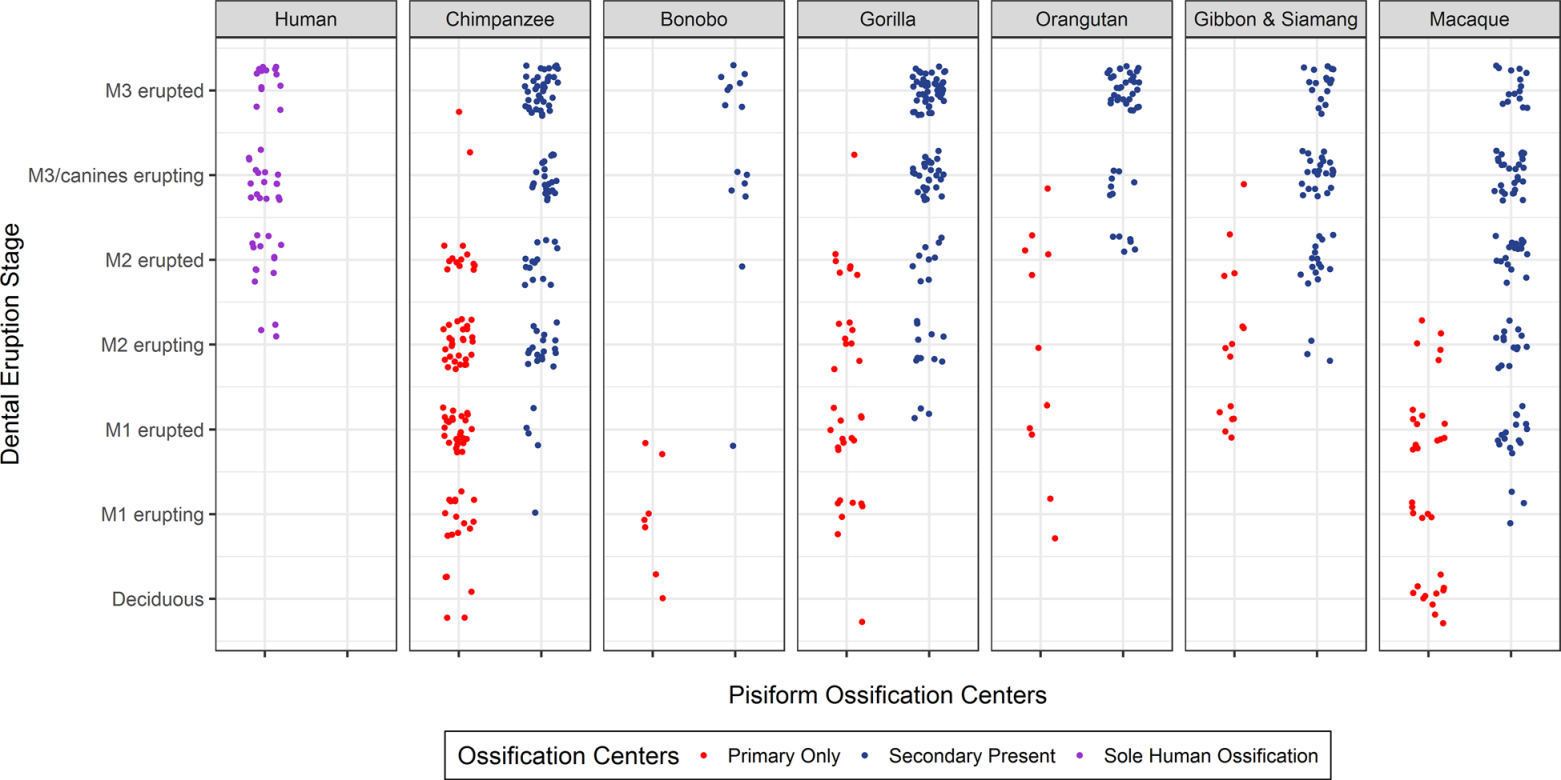
Relationship between dental eruption stages and pisiform ossification number by taxonomic group. Dental eruption stages corresponding to specimens with primary ossification centers only (red) and epiphyses at any stage of ossification (blue). The single human pisiform ossification (purple) is present at the same dental eruption stages as epiphyses of non-human taxa

The earliest examples of non-human pisiform primary ossifications corresponded to dental stages between deciduous and M1 erupted while the earliest unfused pisiforms occurred between M1 erupting and M2 erupted, depending on taxa (Table 3). The earliest identifiable human pisiform ossifications corresponded to M2 erupting, suggesting that they begin ossification when the epiphysis of other taxa is ossifying (Fig. 8). Since human pisiforms lack the distinct ossification stages observed in specimens with two ossification centers, a comparison with pisiform secondary ossifications from non-human groups requires combining unfused, partially fused, and fully fused states to represent all epiphysis developmental stages. In order to further test our hypothesis that human pisiform ossification corresponds to epiphysis ossification of the hominoids, we compared dental eruption stages between species for two conditions: (1) individuals with only a primary ossification center, and (2) individuals with a bony epiphysis (unfused, partially fused, or fully fused). The full human sample was included in both analyses in order to assess identity of the sole human pisiform ossification.

**Table 3:** Dental stage of earliest identifiable ossification centers

Taxonomic group	Pisiform		Calcaneus
	Primary	Unfused	Unfused
Human	M2 erupting		M1 erupting
Chimpanzee	Deciduous	M1 erupting	M1 erupting
Bonobo	Deciduous	M1 erupted	M1 erupting
Gorilla	Deciduous	M1 erupted	Deciduous
Orangutan	M1 erupting	M2 erupted	M1 erupted
Gibbon and siamang	M1 erupted	M2 erupting	M1 erupted
Macaque	Deciduous	M1 erupting	M1 erupting

A Kruskal–Wallis test was performed on dental eruption stage associated with pisiform primary ossification centers from non-human primates and all human pisiform ossifications. This demonstrated a significant difference between taxonomic groups (*X*^2^(6) = 114.190, *N* = 234, *p* = 2.70E−22). We conducted post hoc pairwise Mann–Whitney U tests with a Bonferroni corrected threshold of *p* < 0.00238 (*α* = 0.05/21) to determine which comparisons were significantly different (Table 4). Chimpanzees, gorillas, orangutans, and hylobatids are not significantly different from each other, meaning primary pisiform centers from these groups are ossifying at comparable developmental time points, as determined by dentition. The timing of primary center ossification in macaques is significantly different than all other groups except bonobos, which likely reflects earlier ossification in macaques beginning prenatally. Bonobos also differ significantly from hylobatids, but small sample size limits interpretation of this group. Humans are significantly different from all other groups indicating that the single human pisiform ossification is not comparable to the primary ossification center of other taxa.

**Table 4:** *p*-values for post hoc pairwise Mann–Whitney *U* tests

	Human	Chimpanzee	Bonobo	Gorilla	Orangutan	Hylobatid	Macaque
***Pisiform***							
Human	–	0.884	0.0805	0.0686	1.24E−04*	0.836	0.00102*
Chimpanzee	1.03E−17*	–	0.137	0.0517	1.08E−04*	0.739	4.60E−04*
Bonobo	1.29E−05*	0.00331	–	0.592	0.260	0.0856	8.77E−04*
Gorilla	1.01E−11*	0.967	0.00569	–	0.0108	0.0698	2.95E−04*
Orangutan	6.70E−05*	0.218	0.00800	0.289	–	8.34E−05*	1.71E−10*
Hylobatid	9.07E−07*	0.105	7.05E−04*	0.147	0.978	–	1.57E−04*
Macaque	5.05E−14*	3.91E−05*	0.516	6.14E−04*	0.00225*	7.45E−05*	–
***Calcaneus***							
Human	–	0.419	0.0165	0.413	1.75E−05*	0.877	0.00150*
Chimpanzee		–	0.00705	0.0745	6.40E−07*	0.254	0.00205*
Bonobo			–	0.0832	0.121	0.0214	1.80E−05*
Gorilla				–	9.18E−05*	0.572	3.63E−06*
Orangutan					–	3.31E−05*	1.82E−11*
Hylobatid						–	4.05E−04*
Macaque							–

Mann–Whitney *U* test *p*-values comparing the associations between species of the dental eruption stage of the pisiform primary ossification (top panel, below diagonal), pisiform epiphysis ossification (top panel, above diagonal), and calcaneus epiphysis ossification (lower panel, above diagonal). Humans were included in both pisiform analyses

* Denotes significant *p* values at the Bonferroni corrected *α* = 0.00238

### Human pisiform ossification timing is similar to epiphysis ossification of other hominoids

To further refine the timing of human pisiform development, we compared ossification timing of human pisiforms to the timing of pisiform epiphysis ossification in the other primates. A Kruskal–Wallis test on dental eruption stage associated with pisiform epiphyses in non-human primates and all human pisiforms demonstrated a significant difference between taxonomic groups (*X*^2^(6) = 60.409, *N* = 457, *p* = 3.72E−11). We conducted post hoc pairwise Mann–Whitney *U* tests with a Bonferroni corrected threshold of *p* < 0.00238 (*α* = 0.05/21) to determine which comparisons were significantly different (Table 4). Orangutans are significantly different from all groups except bonobos and gorillas, and macaques are significantly different from all other groups. Secondary centers in macaques seem to appear at slightly earlier dental stages than the hominoids, which is consistent with earlier pisiform primary center ossification when compared to hominoids [49, 52]. Orangutans in our sample tend to have ossifying pisiform epiphyses corresponding to later dental stages than other hominoids. This could be a result of a small sample size for juvenile orangutans, or it could indicate delayed epiphysis ossification and a resulting shorter period of growth as a mechanism for orangutan pisiform reduction. However, more crucially, humans, chimpanzees, bonobos, gorillas, and hylobatids were not significantly different, indicating that the single bone that forms the human pisiform ossifies at the same relative ages as non-human primate pisiform epiphyses.

### Human calcaneal ossification timing is similar to other hominoids

Given that human calcanei have a derived morphology compared to other hominoids, we examined whether timing of calcaneal epiphysis ossification relative to dental age is conserved across species. Calcaneus primary centers begin to ossify during fetal development in all of the studied groups and were found in the youngest specimens examined for this study. The earliest calcaneal epiphysis ossifications were identified ranging from deciduous dentition to M1 erupted, depending on taxa (Table 3). In most instances, this is approximately one dental stage earlier than the earliest identifiable pisiform epiphysis ossification. A Kruskal–Wallis test on dental eruption stage for all specimens with calcaneal epiphysis ossifications found significant differences between taxonomic groups (*X*^2^(6) = 64.729, *N* = 533, *p* = 4.90E−12). Post-hoc pairwise Mann–Whitney *U* tests with a Bonferroni corrected threshold of *p* < 0.00238 (*α* = 0.05/21) reveal that humans, chimpanzees, bonobos, gorillas, and hylobatids were not significantly different (Table 4). These results indicate that calcaneal epiphyses in all hominoids, except orangutans, ossify at comparable dental stages.

## Discussion

### Homology of the human pisiform

The pisiform is a carpal bone with variable morphology across mammalian taxa including hominoids and a notably extreme morphology in humans, but minimal fossil evidence to inform interpretations of these changes. The “pea-shaped” human pisiform is notable because not only it is short, but it also forms from a single ossification center while mammals typically possess two ossification centers with an associated growth plate [4]. We compared ossification patterns and timing across humans, apes, and macaques in order to clarify the homology of the single human pisiform ossification center. In this study, we sought to determine which structure has been lost from the human pisiform in order to determine the homology between the human pisiform and the pisiform ossification centers of other primates and also the relationship with the calcaneus. Additionally, we wanted to know if changes in pisiform ossification timing coincide with changes to calcaneus ossification in humans. The developmental and morphological relationship between pisiforms and calcanei provides an additional comparative approach to study human pisiform evolution by examining variation within and between taxa. We used multiple comparisons to determine the most likely identity of the human pisiform and whether this change has altered calcaneus ossification: (1) morphology of the developing pisiform ossification in mice, humans, and non-human primates; (2) the relationship between pisiform and calcaneus ossification stages; (3) timing of calcaneus ossification stages with respect to dental eruption; and (4) timing of pisiform ossification stages with respect to dental eruption.

Mouse and non-human primates begin pisiform ossification at the dorsal end, form a distinct articular surface for the triquetral at early stages, and possess a subchondral surface prior to fusion between the primary and secondary ossification center. In contrast, the human pisiform ossifies irregularly, forms the palmar portion of the bone first, lacks a distinct articular surface for the triquetral during early ossification, and does not appear to form distinct subchondral surface at its palmar end corresponding to a growth plate. These findings are consistent with reports of normal radiological findings in children [42–45]. The human pisiform sometimes forms from multiple irregular ossification centers [42], a pattern that has been observed previously in human calcaneal epiphyses and gorilla pisiform epiphyses [4, 57]. This developmental trajectory in humans shares more similarities with pisiform epiphysis ossification than it does with the primary center of other species.

Identifying which ossification center was lost from the human pisiform presents a challenge in the absence of a robust hominin fossil record for this bone. Instead, we rely on the developmental and morphological relationship between paralogous structures of the fore- and hind limbs within individuals and comparisons of ossification timing between closely related extant taxa. Ossification initiates earlier in tarsals than carpals in most amniotes [58]. We observe that the earliest identifiable pisiform primary and secondary ossification centers in our sample occur an average of one dental stage later than the comparable structure in the calcaneus (Table 3); however, the majority of non-human primate pisiforms achieve an ossification stage that matches that of the calcaneus within individuals. Thus, even though tarsal development is more advanced than carpals, there is substantial overlap in ossification stages of the pisiform and calcaneus. This includes the majority of individuals with unfused pisiforms (79.4%) also possessing unfused calcanei. The single human pisiform ossification is only found corresponding to calcanei with ossified epiphyses (unfused, partially fused, and fused).

If humans follow the same pattern as non-human primates in our sample, pisiform ossification corresponding with calcaneal epiphysis ossification indicates that human pisiforms more closely align to the developmental stages of the pisiform epiphysis in other primates; however, pisiforms and calcanei have both undergone substantial morphological changes in humans when compared to other hominoids. Therefore, we confirmed that human calcaneal epiphyses ossify at the same molar eruption stages as all other hominoids, except orangutans. Macaques and orangutans differed significantly from all other groups with the macaque appearing to ossify earlier and the orangutan later. Thus, timing of calcaneal epiphysis formation is conserved across most hominoids, including humans. This further supports that the human pisiform most closely corresponds to the epiphysis of other taxa and that changes in human pisiform ossification have not impacted calcaneal epiphysis ossification.

If the human pisiform is homologous to the epiphysis of other species, then we expect it to be present at the same molar eruption stages as epiphysis ossification in non-human pisiforms. If the human pisiform is instead homologous to the primary ossification center of other species, we expect to find it at dental stages comparable to primary ossification centers of other hominoids. The earliest identified human pisiform ossifications occurred while M2 was erupting. This corresponds most closely to ossification in the human calcaneal epiphysis, non-human calcaneal epiphyses, and non-human pisiform epiphyses. Statistical analyses indicate that the human pisiforms are present at the same dental ages as pisiform secondary ossification centers in all hominoids except orangutans, but are significantly different from primary ossification centers in all groups. As with calcanei, macaque pisiform ossification centers appear to form earlier than hominoids, while orangutan epiphysis development appears to be delayed relative to all other groups. Therefore, the sole human pisiform ossification is homologous to the epiphysis in other closely related taxa (Fig. 8).

Homology of the human pisiform with the pisiform epiphysis of other hominoids indicates that the unique human morphology results from the loss of the primary ossification center and the associated growth plate (Fig. 1e-iv). This is in contrast to the only other hominoid with a reduced pisiform, the orangutan, which still retains two ossification centers but may have a shorter period of growth as indicated by delayed epiphysis ossification in both the pisiform and calcaneus relative to other taxa. Pisiforms appear to be highly evolvable across mammals; however, short pisiforms are rare making convergent pisiform reduction between humans and orangutans even more remarkable. The functional role of pisiform reduction is not known, but further studies are warranted to assess commonalities between human and orangutan pisiform reduction. The loss of an ossification center and growth plate represents an exceptional evolutionary event and demonstrates a profound developmental change in the human wrist. Pisiform truncation may constitute one of the more profound developmental changes to the human forelimb since our last common ancestor with chimpanzees.

### Divergence between human forelimbs and hind limbs

The fore and hind limb are paralogous structures that share many aspects of gene expression, regulation, and signaling; however, they are distinguished during embryogenesis by the expression and action of key selector gene transcription factors. The forelimb is characterized by the expression of *Tbx5*, while the hind limb is patterned by the expression of *Pitx1* and *Tbx4*. These produce subsequent downstream effects on other key developmental genes such as the expression of *Hoxc* genes specifically in the hind limb. It has been argued that the extent of shared gene expression in the fore- and hind limbs might produce developmental constraints that must be overcome if selection is to produce divergent phenotypes between the two limbs [47, 59, 60].

More specifically, the pisiform and calcaneus are deemed to be paralogous components within the limbs. This has been confirmed by studies of *Pitx1*. Misexpression of *Pitx1* in the forelimb results in a fusion between the triquetral and pisiform that resembles a calcaneus in both mouse studies and in humans with Liebenberg syndrome [7, 61, 62]. Loss-of-function mutations to *Pitx1* result in a calcaneus that resembles a pisiform [46]. Additionally, the pisiform and calcaneus fall within similar *Hox* expression domains in mice further supporting that these bones are developmentally paralogous structures [8].

The human pisiform and calcaneus have undergone extremely different evolutionary trajectories since our divergence from chimpanzees/bonobos. Not only has the pisiform reduced while the calcaneus expanded, but the entire process of pisiform ossification has been modified with the apparent failure to form the primary center and growth plate. This suggests that selection for each morphology was strong, that the developmental constraints between the two limbs is not particularly intense, or both. Previous work has established that tissue-specific regulatory enhancers can control gene expression with remarkable specificity in a manner that can sculpt skeletal growth [63, 64]. Limb-specific elements have been found that control both Pitx1 and Tbx4 expression in vertebrates [48, 65]. Furthermore, multiple genomic binding cites of Pitx1 that are conserved in both mammals (mouse) and lizards (*Anolis*) have been identified, and these are enriched for genes that play a role in bone and cartilage development [66, 67]. This suggests that variation in the regulatory landscape not only produces divergent limb morphologies but can also differentially target and modify the ossification process between homologous limb structures. It is particularly striking that while the formation of the primary ossification center and growth plate of the pisiform are eliminated, the ossification timing is preserved (i.e., no heterochrony) for the pisiform relative to both calcaneal and pisiform epiphyses of other species.

In fact, the timing of the evolutionary changes in the pisiform and calcaneus do not appear to correspond. Pisiform reduction occurred within the past 3 million years in committed bipeds with reduced arboreal capabilities [68, 69], as evidenced by the elongated *Au. afarensis* pisiform at ~ 3.2 Ma [41] and “pea-shaped” pisiforms described in *Homo neanderthalensis* and *Homo heidelbergensis* [70, 71]. Instead, expanded calcaneal tuberosities were already present within *Au. afarensis* [17]. Thus, changes in the pisiform and calcaneus are both independent developmental and evolutionary transitions.

Given these differences, it is quite possible that the short human pisiform is not related to changes in locomotion but may rather be an adaptation to the evolution of stone tool use. The rarity of short pisiforms in mammals necessitates comparative studies beyond primates to further inform our understanding of the developmental mechanisms and functional implications of pisiform reduction in humans. Such studies may help to clarify whether pisiform reduction is more likely the result of relaxed constraints related to bipedal locomotion or an adaptation to stone tool use. Further studies are also needed to understand the changes in molecular patterning underlying loss of an ossification center and growth plate.

## Conclusion

The human pisiform forms from a single ossification center while most mammals, including apes, form from two. The calcaneus, a paralogous structure to the pisiform, retains two ossification centers in all primates including humans. The single pisiform ossification center in humans develops similarly to the epiphyses of other taxa and it ossifies at the same relative ages as pisiform and calcaneal epiphyses in most apes, and at the same time as the calcaneal epiphysis in humans. These data strongly suggest that the human pisiform is homologous to the pisiform epiphysis of other taxa and that the primary ossification center was lost (Fig. 1e-iv). Loss of the pisiform primary ossification center represents a substantial developmental change that is highly unusual among mammals and likely significant to human evolution.

## Methods

### Human and primate specimens

Non-human primate data were collected at the American Museum of Natural History, New York, NY, USA (AMNH); Anthropological Institute and Museum, University of Zurich, Zurich, Switzerland (AIM); the Hamman-Todd Non-Human Primate Osteological Collection at the Cleveland Museum of Natural History, Cleveland, OH, USA (CMNH); Harvard Museum of Comparative Zoology, Cambridge, MA, USA (MCZ); Smithsonian National Museum of Natural History, Washington, DC, USA (USNM); Powell-Cotton Museum, Birchington, Kent, UK (PCM); and Royal Museum for Central Africa, Tervuren, Belgium (RMCA). Human data were collected from the Hamman-Todd Human Osteological Collection at CMNH.

Specimen inclusion was based on meeting any one of the following criteria: dental eruption was not yet complete, at least one post-cranial epiphysis was unfused or partially unfused, museum notes or materials indicated a specimen as a juvenile or sub-adult, or Powell-Cotton Museum specimens had a maturity index of less than one as determined by Gordon and colleagues [72]. Species, sex, dental eruption pattern, degree of pisiform ossification, and degree of calcaneus ossification were recorded for each specimen. If left and right sides differed, the most advanced ossification or dental eruption stage was used. Fetal specimens were not included in the sample.

### Dental criteria

Dental eruption stage was assessed based on adult molar and canine eruption. Eruption patterns were characterized using methods described by Bolter and Zihlman [55] and Zihlman and colleagues [56]. Adult molars (M1, M2, M3) and canines were classified as “not erupted”, “erupting”, and “erupted”. While most specimens preserved both maxillary and mandibular dentition, scores were still recorded when at least half of one mandible or maxilla was present. When empty sockets were present an attempt was made to locate the associated tooth to determine age. The most advanced dental eruption score for the preserved dental material was used. Partially erupted supernumerary fourth molars were observed in one macaque and two gorilla specimens; these were excluded from analyses that included dental eruption.

### Pisiform and calcaneus ossification criteria

Pisiforms and calcanei were classified based on the number of ossification centers present and degree of fusion between them. Degrees of ossification were categorized as primary ossification only, unfused epiphysis, partially fused epiphysis, or fully fused epiphysis. Occasionally we identified fully cartilaginous pisiforms; these were studied for descriptive purposes but were not included in statistical analyses. Unfused specimens were defined as those with no visible fusion between two ossification centers. Partially fused specimens were defined as those with any amount of connection along the outer edge of the primary and secondary ossification centers for which a visible demarcation of the two centers was visible. We considered a specimen to have a fully fused, adult morphology when the epiphyseal line was no longer detectible. Most specimens were visually inspected; however, radiographs were obtained when possible for specimens with dried soft tissue elements obscuring the pisiform or calcaneus. We suspect that unfused pisiform epiphysis ossifications were sometimes not preserved in museum specimens and are, therefore, underrepresented in this sample and consequently, the number of primary ossification centers without epiphysis ossifications is likely inflated. In some cases, ossification centers were encased in cartilaginous or ligamentous material, providing more certainty that the epiphysis ossification was not formed. Specimens represented a full range of ossification stages for both the primary and secondary ossification centers, and we rely on multiple measures in our analysis including earliest appearance of each ossification center in addition to timing based on dental eruption. Additionally, statistical analyses of secondary ossifications are significantly different from primary ossifications despite increased overlap due to the likely inflated number of primary ossification centers.

### Mouse samples

Histological analysis was performed on paraffin-embedded forepaws from FVB/NJ mice euthanized at post-natal days P4–P30. Tissue was fixed in 4% paraformaldehyde in 1× PBS, decalcified in 10% EDTA, dehydrated, and embedded in paraffin following standard protocols. Tissue sections were stained with Safranin-O and Fast Green to visualize cartilage and bone. A microCT scan with 7 μm resolution was obtained for a 1-month-old mouse using a GE v|tome|x housed at Pennsylvania State University’s Applied Research Laboratory. Images of microCT data for this paper were generated using Dragonfly software (Object Research Systems Inc), Version 4.0 [73]. IACUC approval was obtained and institutional protocols were followed for housing and euthanasia.

## Data Availability

The datasets used and/or analyzed during the current study are available from the corresponding author on reasonable request.
